# Childhood hunger and cognitive impairment in later life among older adults in China: a nationwide case-control study using target maximum likelihood estimation

**DOI:** 10.7189/jogh.16.04317

**Published:** 2026-10-30

**Authors:** Wei Wang, Yingtian Wang, Xinru Hu, Fangyi Chen, Qian Yu, Xirui Qiu, Fan Yang, Li Liu, Xiaoxiao Wang

**Affiliations:** 1 GCP Centre, Affiliated Hospital of Nanjing University of Chinese Medicine, Nanjing, China; 2 School of Nursing, Nanjing University of Chinese Medicine, Nanjing, China; 3 Department of Public Health, School of Medicine, Nanjing University of Chinese Medicine, Nanjing, China; 4 Department of Science and Technology, Affiliated Hospital of Nanjing University of Chinese Medicine, Nanjing, China; 5 Department of Respiratory and Critical Medicine, Affiliated Hospital of Nanjing University of Chinese Medicine, Nanjing, China; 6 School of Public Health, Nanjing Medical University, Nanjing, China; 7 Clinical Research Institute, Affiliated Hospital of Nanjing University of Chinese Medicine, Nanjing, China

**Keywords:** childhood hunger, cognitive impairment, adverse childhood experience, case-control study, target maximum likelihood estimation, E-value

## Abstract

**Background:**

Early life nutritional deprivation is associated with poor health outcomes in later life, such as cognitive impairment. Since many older Chinese adults experienced frequent hunger during childhood, we aimed to estimate its impact on late-life cognitive impairment in older adults.

**Methods:**

We included participants aged 65–99 years from the 2017–2018 wave of the Chinese Longitudinal Healthy Longevity Survey, who completed the Mini-Mental State Examination (MMSE) and reported whether they experienced childhood hunger. We classified participants based on their MMSE scores into a cognitive impairment group (case group) and a control group. We used an unmatched case-control design, with unconditional logistic regression and case-control weighted target maximum likelihood estimation (CCW-TMLE), to assess the impact of childhood hunger on late-life cognitive impairment, and conducted sensitivity analyses to evaluate robustness of the association between them.

**Results:**

Among 9,297 participants, 1,108 (11.92%) had cognitive impairment. Childhood hunger was more prevalent in the cognitive impairment group (76.62% *vs.* 69.48%, *P* < 0.001). In the full model, childhood hunger was significantly associated with a higher prevalence of cognitive impairment (odds ratio (OR) = 1.229; 95% confidence interval (CI) = 1.044–1.447, *P* = 0.013) compared to the non-childhood hunger group. CCW-TMLE also found that the childhood hunger group had a higher prevalence of later-life cognitive impairment (risk ratio (RR) = 1.167; 95% CI = 1.046–1.303; OR = 1.224, 95% CI = 1.063–1.410). The results of sensitivity analyses supported the robustness of the impact.

**Conclusions:**

Childhood hunger was associated with a higher prevalence of cognitive impairment in later life. The findings suggest that improving childhood nutrition and food security might be linked to a lower burden of late-life cognitive impairment, particularly in economically disadvantaged populations. However, further research is needed to establish causality.

There were 164.5 million individuals aged ≥65 years in China in 2019 [1]. With the ongoing growth of the older population, age-related conditions (*e.g.* cognitive impairment, mental disorders) have become increasingly prevalent. In a large Chinese cross-sectional study, the prevalence of mild cognitive impairment was 15.5%, with an overall dementia prevalence of 6.0%, representing an estimated 15.07 million cases in adults aged ≥60 years [2]. Cognitive impairment is widely regarded as a transitional state between normal cognitive ageing and dementia [3]. To date, no pharmacological intervention has been proven to slow or halt the progression from mild cognitive impairment to dementia [4]. Thus, accurately identifying modifiable factors for cognitive decline is essential to formulate effective public health strategies and promote healthy ageing in China.

Previous studies have identified several modifiable risk factors for cognitive decline, including educational attainment in early life, depression, smoking, atrial fibrillation, dyslipidaemia, and benzodiazepine use in mid- to late- life [5–7]. Growing evidence suggests that the origins of dementia may extend back to early life [8], and a disadvantaged early-life environment appears to elevate the risk of dementia and cognitive decline in later years [6,9–11]. A meta-analysis reported that food deprivation, leg length, and education were associated with dementia risk, with individuals who experienced childhood food deprivation exhibiting nearly twice the risk of cognitive impairment compared to those who did not [12]. Furthermore, a European longitudinal analysis revealed that experiencing hunger between ages 0–4 years was particularly detrimental to cognitive function in later life [10].

Notably, many older adults currently over the age of 65 in China experienced hunger, poverty, and social instability during their childhood or adolescence [13]. This underscores the importance of investigating whether childhood hunger among older Chinese adults has significant long-term cognitive consequences. A study using data from the 2002 and 2005 waves of the Chinese Longitudinal Healthy Longevity Survey (CLHLS) examined the relationship between childhood nutritional deprivation and cognitive impairment in older adults [14]. However, the study had several methodological limitations: failing to adjust for educational level in cognitive assessment; including inappropriate variables in regressions that were not true confounders; potential collinearity from simultaneously modelling childhood hunger and anthropometric measures as proxies for nutritional deprivation; and use of an inappropriate statistical model, for which a conditional survival analysis would have been more suitable.

To address these limitations, we designed a case-control study based on the 2017–2018 wave of CLHLS to explore association between a known health outcome and retrospectively reported prior exposures. We used a doubly robust estimator, namely case-control weighted target maximum likelihood estimation (CCW-TMLE) [15,16], to clarify the relationship between childhood hunger and late-life cognitive impairment.

## METHODS

Here, we report a secondary analysis of an open-access dataset, conducted in accordance with the Journal of Global Health’s Guidelines for Reporting Analyses of Big Data Repositories Open to the Public (GRABDROP) (Table S1 in the **Online Supplementary Document**) [17].

### Study design and participants

We designed an unmatched case-control study based on cognitive impairment among participants aged 65–99 years in the CLHLS 2017–2018 data and their exposure to childhood hunger. We also designed a matched case-control study based on the same dataset after 1:1 propensity score matching (PSM) in sensitivity analyses.

We included 15,874 participants, of whom 12,411 were newly recruited in the 2017–2018 wave, and 3,463 were carried over from previous waves. We excluded participants who had incomplete responses on the Chinese version of the Mini-Mental State Examination (MMSE), or did not answer the question about whether they ‘often went to bed hungry as a child’ (**Figure 1**).

**Figure 1:**
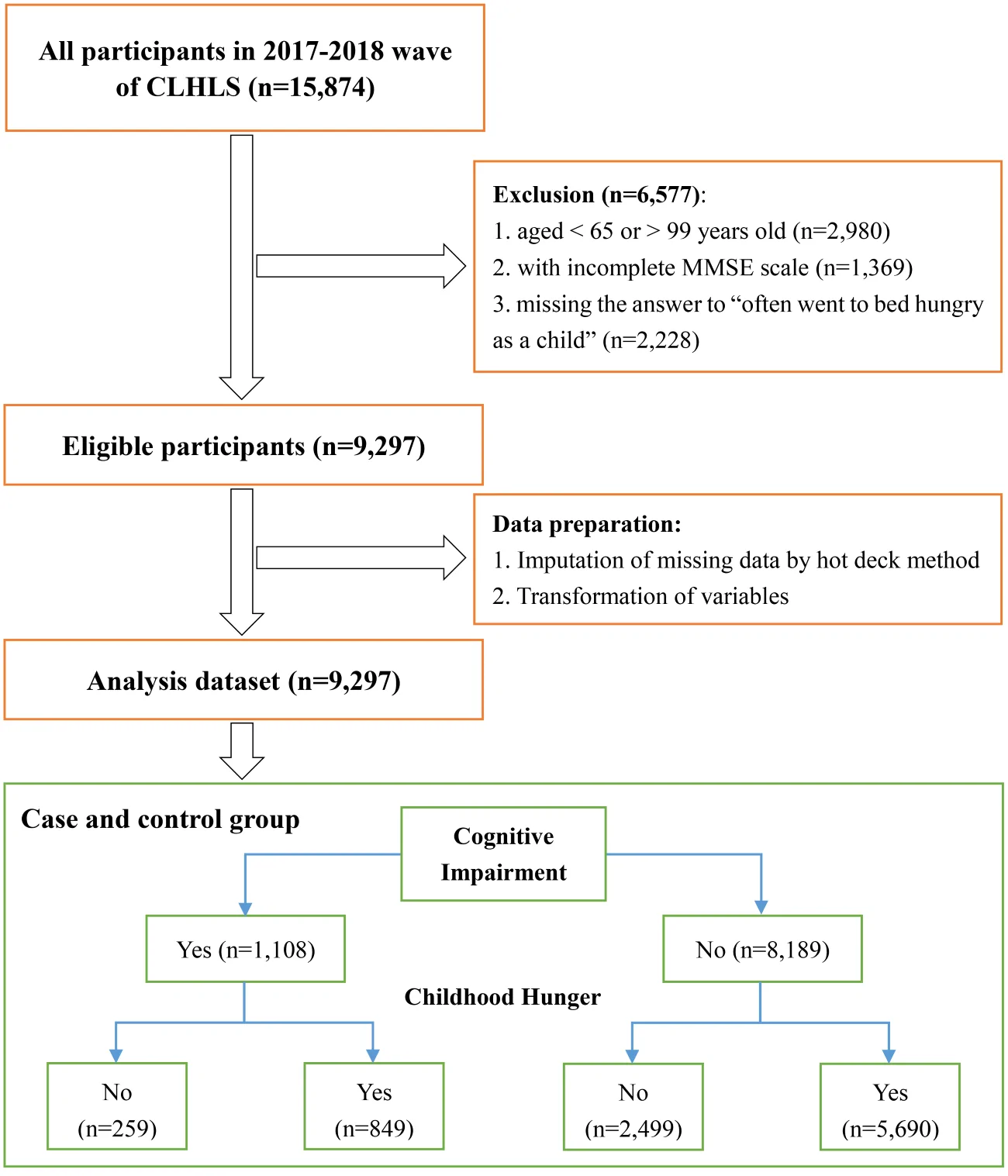
The screening process of eligible participants.

CLHLS was a longitudinal healthy longevity survey among Chinese people aged 65 years and over. The CLHLS included eight subsequent waves of surveys conducted in 23 provinces, municipalities and autonomous regions, in addition to the first survey in 1998. Detailed information about CLHLS is shown on the official website of Peking University Centre for Healthy Ageing and Development.

### Measurement of exposure

Childhood hunger, caused by poverty and other reasons, is considered an adverse childhood experience and a proxy for nutritional deprivation affecting health in later life [14,18]. In the 2017–2018 CLHLS, participants were asked, ‘Did you ofteen go to bed hungry as a child?’, and responded ‘Yes’ or ‘No’, indicating whether they experienced childhood hunger.

Additionally, arm length and knee height are common anthropometric measures that can serve as indicators of childhood nutritional deprivation [14]. These measures were classified as abnormal if they fell below the sex-specific 10th percentile and as normal otherwise. In a sensitivity analysis, we used abnormal arm length or knee height as an objective proxy for childhood hunger.

### Assessment of outcome

To account for the influence of educational attainment on cognitive function, we defined cognitive impairment using the Chinese version of MMSE (Table S2 in the **Online Supplementary Document**) cut-off scores according to years of schooling: below 18 for participants with no formal education, below 21 for those with 1–6 years of schooling, and below 25 for those with more than 6 years of schooling [19].

### Covariates

Based on the definition of confounders and the direction of the causal pathways from confounders to the exposure and outcome in directed acyclic graphs (Figure S1 in the **Online Supplementary Document**), we identified the following childhood-related variables as candidate confounders: sex (male or female), race (Han or minority), the region of birth (eastern region, the central region, northeast region or western region), the residence of birth (urban or rural), the decade of birth (1910s–1920s, 1930s, 1940s or 1950s), main source of water drank in childhood (nature water or tap water), drink in childhood (yes or no), physical labour in childhood (yes or no), received schooling in childhood (yes or no), mother received schooling (yes or no), father received schooling (yes or no), father had stable job in participants’ childhood (yes or no), lost mother in childhood (yes or no), and lost father in childhood (yes or no). We extracted these variables directly from the CLHLS or derived them from relevant survey information. They reflect participants’ inherent characteristics, regional and household socioeconomic conditions, childhood experiences and family educational backgrounds, *etc*. (Table S2 in the **Online Supplementary Document**).

### Statistical analysis

We first analysed the extent of missing data (Table S3 in the **Online Supplementary Document**) and described how we handled variables with missing values. We excluded covariates with 30% or more missing data and imputed missing values for covariates with less than 30% missing data using the hot-deck method, with a random seed of 316.

We described continuous variables as means and standard deviations (SDs) or medians and quartiles (Q_1_, Q_3_) according to the variable’s normal distribution, and compared the differences between the case group and control group using a *t*-test or Wilcoxon rank-sum test. We described categorical variables by frequency (n) and percentage (%), and compared the two groups using the χ^2^ test. We considered candidate confounders with *P* < 0.05 in the between-group comparison as confounders.

We estimated the impact of childhood hunger on cognitive impairment using hierarchical regression analysis based on unconditional logistic regressions. We also estimated risk ratio (RR), odds ratio (OR) and risk difference (RD) using CCW-TMLE, a weighted analysis, that we calculated as follows [15]: assuming that the weights assigned to the unmatched case and control groups are W1 and W0, and the prevalence of the disease in the target study population is PY1, then W1 equals PY1, and W0 equals (1-PY1)/J, where J is the ratio of the number of controls (n = 8,189) to the number of cases (n = 1,108) in the study sample. As indicated by the study of Chen *et al.* using CLHLS data [20], the prevalence of cognitive impairment among Chinese older adults in 2018 was 24.9%. Therefore, W1 is 0.249, and W0 is 5.550. Notably, super learners [21] with four common base learners, including generalised linear models (SL.glm), generalised additive models (SL.gam), stepwise regression based on Akaike’s Information Criterion (SL.stepAIC) and elastic net regression (SL.glmnet) were implemented in the CCW-TMLE.

We first performed sensitivity analysis using 1:1 PSM with a calliper width of 0.05. Second, we conducted a matched case-control study and conditional logistic regressions based on the dataset after PSM. Third, we re-executed a multivariable unconditional logistic regression and a multivariable-adjusted CCW-TMLE, in which the exposure was replaced by a binary variable indicating normal *vs.* abnormal arm length or knee height. Fourth, we performed the multivariable unconditional logistic regression and CCW-TMLE based on a dataset imputed by a random forest algorithm. Fifth, we re-implemented unconditional logistic regression and CCW-TMLE, adjusting for candidate confounders with *P* < 0.20 between-group comparison. Sixth, because participants in this study were aged 65–99 years, whereas those in the study by Chen *et al.* [20] were aged 65-105, the prevalence of cognitive impairment in the target population was reduced by 10% (to 22.41%), and CCW-TMLE was repeated. Seventh, we used a cut-off score of 18 on the MMSE as the classification threshold, regardless of educational level, and refitted the multivariable unconditional logistic regression and the CCW-TMLE [22,23]. Finally, we calculated the E-value [24] to assess the robustness of the observed association to potential unmeasured confounding.

We performed all statistical analyses using *R*, version 4.3.1 (R Core Team, Vienna, Austria) with packages VIM, MatchIt, survival, tmle, SuperLearner and randomForest. The level of statistical significance was set at *P* ≤ 0.05.

## RESULTS

### Characteristics of eligible participants

We enrolled a total of 9,297 participants into the analysis dataset, including 1,108 (11.92%) participants with cognitive impairment and 8,189 (88.08%) without cognitive impairment (**Figure 1**). The mean age of participants at survey was 81.14 (SD = 9.57) years; 47.38% of all were male, 18.11% of all were born in urban areas, more than 90% of all were born in the first half of the 20th century, 12.90% of all participated in physical labour in childhood, and 57.75% of all received schooling in childhood.

The proportion of childhood hunger in the case group was higher than that of the control group (76.62% *vs.* 69.48%, *P* < 0.001). Meanwhile, the constituent ratios of sex (*P* < 0.001), the decade of birth (*P* < 0.001), received schooling in childhood (*P* < 0.001), mother received schooling (*P* = 0.004), father received schooling (*P* < 0.001), father had a stable job in participants’ childhood (*P* = 0.002) and lost mother in childhood (*P* = 0.033) between case group and control group showed significant difference (**Table 1**).

**Table 1:** The description of characteristics and comparison of those between the case group and control group

		Cognitive impairment			
	**Total (n = 9,297)**	**No (n = 8,189)**	**Yes (n = 1,108)**	***Z/χ^2^*/*t***	***P*-value**
**MMSE, Mdn (Q_1_, Q_3_)**	28.00 (24.00, 30.00)	29.00 (26.00, 30.00)	13.00 (7.00, 17.00)	53.772	<0.001
**Childhood hunger**				23.853	<0.001
Yes	6,539 (70.33)	5,690 (69.48)	849 (76.62)		
No	2,758 (29.67)	2,499 (30.52)	259 (23.38)		
**Arm length or knee height**				23.489	<0.001
Normal	7,895 (85.68)	7,017 (86.33)	878 (80.85)		
Abnormal	1,319 (1319)	1,111 (13.37)	208 (19.15)		
**Age, x̄ (SD)**	81.14 (9.57)	79.96 (9.21)	89.90 (7.35)	−40.877	<0.001
**Sex**				22.493	<0.001
Male	4,405 (47.38)	3,954 (48.28)	451 (40.70)		
Female	4,892 (52.62)	4,235 (51.72)	657 (59.30)		
**Race**				1.563	0.211
Han	8,795 (94.60)	7,738 (94.49)	1,057 (95.40)		
Minority	502 (5.40)	451 (5.51)	51 (4.60)		
**The region of birth**				2.004	0.572
Eastern region	4,502 (48.42)	3,982 (48.63)	520 (46.93)		
The central region	2,109 (22.68)	1,844 (22.52)	265 (23.92)		
Northeast region	403 (4.33)	359 (4.38)	44 (3.97)		
Western region	2,283 (24.56)	2,004 (24.47)	279 (25.18)		
**The residence of birth**				0.945	0.331
Urban	1,684 (18.11)	1,495 (18.26)	189 (17.06)		
Rural	7,613 (81.89)	6,694 (81.74)	919 (82.94)		
**The decade of birth**				1,069.490	<0.001
1910s–1920s	2,691 (28.94)	1,918 (23.42)	773 (69.77)		
1930s	2,810 (30.22)	2,576 (31.46)	234 (21.12)		
1940s	2,800 (30.12)	2,717 (33.18)	83 (7.49)		
1950s	996 (10.71)	978 (11.94)	18 (1.62)		
**Main source of water drunk in childhood**				0.541	0.462
Nature water	8,401 (90.36)	7,393 (90.28)	1,008 (90.97)		
Tap water	896 (9.64)	796 (9.72)	100 (9.03)		
**Drink in childhood**				0.037	0.848
No	9,234 (99.32)	8,134 (99.33)	1,100 (99.28)		
Yes	63 (0.68)	55 (0.67)	8 (0.72)		
**Physical labour in childhood**				0.893	0.345
No	8,098 (87.10)	7,123 (86.98)	975 (88.00)		
Yes	1,199 (12.90)	1,066 (13.02)	133 (12.00)		
**Received schooling in childhood**				203.010	<0.001
No	3,928 (42.25)	3,240 (39.57)	688 (62.09)		
Yes	5,369 (57.75)	4,949 (60.43)	420 (37.91)		
**Mother received schooling**				8.127	0.004
No	8,792 (94.57)	7,724 (94.32)	1,068 (96.39)		
Yes	505 (5.43)	465 (5.68)	40 (3.61)		
**Father received schooling**				54.806	<0.001
No	7,310 (78.63)	6,344 (77.47)	966 (87.18)		
Yes	1,987 (21.37)	1,845 (22.53)	142 (12.82)		
**Father had a stable job in participants’ childhood**				9.338	0.002
Yes	1,981 (21.31)	1,784 (21.79)	197 (17.78)		
No	7,316 (78.69)	6,405 (78.21)	911 (82.22)		
**Lost mother in childhood**				4.521	0.033
No	8,404 (90.39)	7,422 (90.63)	982 (88.63)		
Yes	893 (9.61)	767 (9.37)	126 (11.37)		
**Lost father in childhood**				2.677	0.102
No	7,990 (85.94)	7,020 (85.72)	970 (87.55)		
Yes	1,307 (14.06)	1,169 (14.28)	138 (12.45)		

Mdn – median, MMSE – Mini-Mental State Examination, SD – standard deviation, x̄ – mean

*Presented as n (%) unless specified otherwise.

### Association between childhood hunger and cognitive impairment

The crude OR from Model 1 provided a preliminary indication that the association between childhood hunger and later-life cognitive impairment was statistically significant (OR = 1.440; 95% confidence interval (CI) = 1.243–1.667, *P* < 0.001). Thereafter, we found that after adjustment for confounders, the association between exposure and outcome was also statistically significant. Model 4 (**Table 2**; Table S4 in the **Online Supplementary Document**) adjusting for all confounders suggested that the prevalence of cognitive impairment in participants exposed to childhood hunger increased by 22.9% compared to participants who were not exposed to childhood hunger (adjusted OR = 1.229; 95% CI = 1.044–1.447, *P* = 0.013). The RR and OR estimated by CCW-TMLE (**Table 3**) indicated higher odds and risk of cognitive impairment in later life for the childhood hunger group compared to the non-childhood hunger group (RR = 1.167; 95% CI = 1.046–1.303; and OR = 1.224; 95% CI = 1.063–1.410). The RD (**Table 3**) for the childhood hunger group was 36.36 per 1000 population (95% CI = 11.70–61.02).

**Table 2:** The association between childhood hunger and cognitive impairment in later life*

Childhood hunger	OR (95% CI)	*P*-value	E-value
Model 1			
*No*	ref		
*Yes*	1.440 (1.243 − 1.667)	<0.001	2.236
Model 2			
*No*	ref		
*Yes*	1.235 (1.053 − 1.449)	0.009	1.774
Model 3			
*No*	ref		
*Yes*	1.234 (1.049 − 1.453)	0.011	1.771
Model 4	ref		
*No*			
*Yes*	1.229 (1.044 − 1.447)	0.013	1.760

CI – confidence interval, OR – odds ratio, ref – reference

*Model 1: childhood hunger; Model 2: childhood hunger, the decade of birth, sex and received schooling in childhood; Model 3: childhood hunger, the decade of birth, sex, received schooling in childhood, mother received schooling, father received schooling and father had a stable job in participants’ childhood; Model 4: childhood hunger, the decade of birth, sex, received schooling in childhood, mother received schooling, father received schooling, father had a stable job in participants’ childhood and lost mother in childhood.

**Table 3:** The association between childhood hunger and cognitive impairment in later life using the CCW-TMLE method*

Causal effect measures	Estimate	95% CI	*P*-value	E-value
RR	1.167	1.046–1.303	0.006	1.609
OR	1.224	1.063–1.410	0.006	1.748
RD (‰)	36.36	11.70–61.02	0.004	-

CCW-TMLE – case-control weighted target maximum likelihood estimation, CI – confidence interval, OR – odds ratio, RD – risk difference, RR – risk ratio

*Confounders: the decade of birth, sex, received schooling in childhood, mother received schooling, father received schooling, father had a stable job in participants’ childhood and lost mother in childhood.

### Sensitivity analyses

The results of sensitivity analyses were consistent with results of the main analysis. First, the proportion of childhood hunger in the case group was higher than that of the control group (76.62% *vs.* 72.47%, *P* = 0.025) in the dataset after PSM (Table S5 in the **Online Supplementary Document**). Second, the conditional logistic regression suggested the odds of being in the childhood hunger group were 1.288 times higher than in the non-childhood hunger group (OR = 1.288; 95% CI = 1.047–1.583). Third, after excluding participants with missing data on both arm length and knee height, we included 9,214 participants in the analysis, of whom 1,319 (14.32%) were likely to have experienced nutritional deprivation. The proportion of abnormal arm length or knee height was significantly higher in the case group than that in the control group (19.15% *vs.* 13.67%, *P* < 0.001) (**Table 1**). Participants with abnormal arm length or knee height had higher odds of cognitive impairment than those with normal measurements (Tables S6 and S7 in the **Online Supplementary Document**). Fourth, the analyses based on the dataset imputed by the random forest algorithm also suggested childhood hunger might be a risk factor for cognitive impairment in later life (Tables S8 and S9 in the **Online Supplementary Document**). Fifth, unconditional logistic regression showed a significant association, and CCW-TMLE adjusting for confounders, with *P* < 0.20 in between-group comparison (Tables S10 and S11 in the **Online Supplementary Document**). Sixth, after the prevalence of cognitive impairment in the target population was lowered to 22.41%, the CCW-TMLE (Table S12 in the **Online Supplementary Document**) also indicated an association between childhood hunger and increased odds of cognitive impairment in later life (RD = 34.73; 95% CI = 11.74–57.73; and RR = 1.179; 95% CI = 1.052–1.322; and OR = 1.232; 95% CI = 1.068–1.422). Seventh, after we reclassified participants into case and control groups using an MMSE cut-off score of 18 points, the CCW-TMLE results were consistent with the main findings (Tables S13 and S14 in the **Online Supplementary Document**). Finally, the E-value (**Table 2**, **Table 3**) suggested that unmeasured confounding would need to be associated with both childhood hunger and cognitive impairment by an RR or OR of at least 1.609 to 2.236 to fully explain the observed association.

## DISCUSSION

### Main findings

We found that childhood hunger may increase the odds of cognitive impairment in later life by approximately 20%, and that eliminating childhood hunger could reduce the number of cognitive impairment cases by about 36 per 1,000 older adults. These exploratory findings may add one piece of evidence to the association between childhood nutritional deprivation and later-life cognitive function, and could serve as a reference for international non-governmental organisations and regional agencies in implementing child rights protection and in identifying older adults with a history of childhood hunger as a high-risk group for cognitive impairment.

### Interpretation and comparison

The findings are congruent with previous research demonstrating a negative impact of childhood hunger on cognitive functions. For example, malnutrition during childhood in India can impede the cognitive development of children [25,26]. Early-life experiences of hunger predict lower performance in delayed recall and language fluency during old age in Europe [27]. Our results are similar to those of previous studies, suggesting that adversity in early life has lasting effects on brain structure and cognitive function in old age [28,29]. Cui *et al.* provided evidence that childhood hunger tends to have more detrimental cognitive effects on women [30]. Similarly, previous studies on famine reported that exposure to famine during mid- to late-childhood is associated with poorer performance on the Stroop Color and Word Test [31]. Also, severe famine has a significant impact on the cognitive development of children aged 0 to 2 years who have experienced hunger [32].

Little is known about the exact mechanism between food deprivation during childhood and cognitive decline in later life. It is widely recognised that adequate nutrition is essential for normal brain development, and early life is a pivotal period for this development. At the same time, inadequate nutrition also increases the risk of neurodevelopmental and cognitive deficits. Now, there is ample evidence that early-life adversity in the form of malnutrition leads to impaired cognitive function, which is associated with disruption of hippocampal neurogenesis and altered synaptic plasticity [9,33,34]. In recent years, nutrients such as B vitamins and omega-3 fatty acids have been extensively studied in terms of brain development and function [35]. Prado *et al.* [36] reviewed the evidence for the effects of specific nutrient deficiencies during early development on key neurodevelopmental processes. Childhood undernutrition may reduce the number of brain cells and neurons, which in turn affects normal brain development and even leads to hypoplasia. This condition could elevate the risk of cognitive dysfunction. Moreover, malnutrition has a lasting impact on glial fibrillary acidic protein expression in astrocytes [37].

### Practical implications

Drawing on these findings, we propose several tentative implications for consideration from a global public health perspective.

First, international organisations focused on child welfare and health should consider systematically compiling evidence from studies on famine or childhood hunger and adverse adult outcomes. Such efforts would produce more conclusive findings to guide targeted economic and nutritional assistance for food-insecure low- and middle-income countries, thereby helping reduce the global cognitive impairment burden. Second, older adults with a history of childhood hunger may be regarded as a potentially vulnerable subgroup with a slightly elevated prevalence of cognitive impairment. Where resources permit, targeted early screening and intervention programs could be considered for this population, although such efforts should be proportionate to the current evidence base. Third, the WHO and other national or regional bodies could consider developing life-course information surveillance systems or upgrading current research-based databases to include more adverse childhood experiences that are relevant to health. This infrastructure would underpin future cohort studies with enhanced causal inference, thereby advancing knowledge of the lifelong health consequences of childhood adversity.

### Strengths and limitations

The primary strengths of this study are as follows. First, we used a large, nationwide sample of community-dwelling older adults in China, ensuring a large sample size and enhancing the representativeness of the study population. Second, because information on older adults’ childhood experiences was collected through oral interviews, we adopted a case-control study design, which we considered more appropriate. Third, CCW-TMLE combines case-control weighting and TMLE to estimate exposure–outcome associations robustly from a case-control study. It is particularly well suited for assessing such associations in the context of rare diseases or large-scale observational studies [38]. Lastly, multiple sensitivity analyses were conducted to verify the robustness of results.

To properly interpret these findings, limitations should be acknowledged. First, we obtained data on childhood hunger from self-report questionnaires. Older participants and those with cognitive impairment may recall childhood adversities differently from other participants, potentially introducing recall bias and exposure misclassification. Second, restricting cases and controls to surviving elderly may introduce survival bias, since severe childhood hunger could have caused earlier deaths, thus systematically excluding those individuals. This selective survival biases the childhood hunger and cognitive impairment association, potentially toward the null or even underestimation. Third, although the Chinese MMSE is widely used for screening, it cannot replace formal clinical diagnosis, so some misclassification against gold-standard criteria may remain. Fourth, although CCW-TMLE can theoretically provide robust estimates of the exposure–outcome association, this study remains a database-based observational case-control study, which cannot guarantee that the assumptions for causal inference (*e.g.* no unmeasured confounding) were satisfied. Fifth, lacking data on age and duration of hunger onset, we could not further examine the dose-response association between early nutritional deprivation and later cognitive impairment. Finally, due to design constraints, we did not address sex moderation, nor the mediating effects of adult socioeconomic status or physical health. These warrant further investigation using birth cohorts, such as the Avon Longitudinal Study of Parents and Children, for more scientifically rigorous exploration.

## CONCLUSIONS

In this study, frequent childhood hunger was associated with a higher prevalence of cognitive impairment in later life. These findings highlight the importance of children’s living conditions and food security, particularly in economically disadvantaged areas, as improving these factors may correlate with better cognitive outcomes in old age.

## Additional material


Online Supplementary Document

## Data Availability

**Data availability:** The datasets that support this article are publicly available from the project of the CLHLS at https://opendata.pku.edu.cn/dataverse/CHADS.
