# Supplementary material for: Childhood hunger and cognitive impairment in later life among older adults in China: a nationwide case-control study using target maximum likelihood estimation
Source: J Glob Health. 2026 Oct 30;16:04317. doi: 10.7189/jogh.16.04317 (PMC13721837; doi:10.7189/jogh.16.04317)
Supplement: Supplementary file 1 [file jogh-16-04317-s001.pdf]

Supplement to: Wang W, Wang Y, Hu X, Chen F, Yu Q, Qiu X, Yang F, Liu L, Wang X. Childhood hunger and cognitive impairment in later life among older adults in China: a nationwide case-control study using target maximum likelihood estimation. J Glob Health. 2016;16:04317.

Table S1. Adherence to JoGH's GRABDROP guidelines items
Table S2. The names, labels and transformation of variables in the study.
Table S3. The description of variables with missing value.
Table S4. Detailed information about hierarchical regressions.
Table S5. The comparison of characteristics between case group and control group after 1:1 propensity score matching.
Table S6. The association between arm length or knee height and cognitive impairment based on multivariable unconditional logistic regressions
Table S7. The association between arm length or knee height and cognitive impairment based on CCW-TMLE
Table S8. The multivariable unconditional logistic regression based on dataset imputed by random forest algorithm.
Table S9. The CCW-TMLE method based on dataset imputed by random forest algorithm.
Table S10. The multivariable unconditional logistic regression adjusting furtherly an additional confounder.
Table S11. The CCW-TMLE method adjusting furtherly an additional confounder..
Table S12. The CCW-TMLE method based on a target population prevalence of cognitive impairment set to 22.41%
Table S13. The multivariable unconditional logistic regression using an MMSE cutoff of 18 to redefine cognitive impairment.
Table S14. The CCW-TMLE method using an MMSE cutoff of 18 to redefine cognitive impairment.
Figure S1. The simplified causal directed acyclic graphs showed the relationship and directions of path among exposure, confounders and outcome.

Table S1. Adherence to JoGH's GRABDROP guidelines items

**JoGH guideline items**
1. Please list all papers published by each co-author in previous 3 years that were based on secondary analysis of a big data repository
[1] **Xiaoxiao Wang** ,**Xinru Hu**, **Fangyi Chen**, **Qian Yu**, **Yingtian Wang**, **Xirui Qiu**, **Fan Yang**, **Wei Wang**. Association between childhood hunger and depression or anxiety in later life: Insights from two case-control studies. Psychiatry Res. 2025;348:116475. doi:10.1016/j.psychres.2025.116475<br>[2] **Yingtian Wang**, Haining Qi, **Xinru Hu**, **Qian Yu**, Manshu Yu, **Fan Yang**, **Xirui Qiu**, **Fangyi Chen**, Ye Ding, Meng Jiang, **Xiaoxiao Wang**, **Liu Li**, **Wei Wang**. The interaction between activities of daily living and psychological resilience on all-cause mortality and cognitive impairment among Chinese older adults: A cohort study based on CLHLS. Arch Gerontol Geriatr. 2026;140:106047. doi:10.1016/j.archger.2025.106047<br>[3] **Xiaoxiao Wang**, **Wei Wang**, Hanling Zeng, **Xinru Hu**, **Fangyi Chen**, Li Shen, Jing Tao. Molecular structure of polysaccharide mediated autophagy markers KIF23 and PRC1 proteins and their regulatory role in triple negative cancer through the p53 signaling pathway. Int J Biol Macromol. 2025;291:139155. doi:10.1016/j.ijbiomac.2024.139155<br>[4] **Wei Wang**, Hui Wang, **Xinru Hu**, **Qian Yu**, **Fangyi Chen**, **Xirui Qiu**, **Xiaoxiao Wang**. The Association Between Posting WeChat Moments and the Risk of Depressive Symptoms Among Middle-Aged and Older Chinese Adults: Prospective National Cohort Study. JMIR Public Health Surveill. 2025;11:e62730. doi:10.2196/62730<br>[5] Yifan Wang, Tianchen Qi, Xiaoli Wu, Chunhao Li, Boyan Zeng, Peiqi Huang, Dan Liu, Shuang Liang, **Fan Yang**, Feifei Xu, Chang Su, Bo Zhang, Juncheng Dai. Ultra-processed foods, biological ageing, and all-cause mortality risk: a prospective cohort study using 172,225 participants from UK Biobank. Geroscience. 2025;47(5):6599-6612. doi:10.1007/s11357-025-01810-7<br>[6] Yifan Wang, Yue Sun, **Fan Yang**, Musu Li, Tianchen Qi, Zixuan Lu, Qian Wang, Qingyin Bu, Longhu Sun, Hongmei Wo, Yang Zhao, Honggang Yi, Juncheng Dai. ProtPhenoAge: Integrating plasma proteomics to predict Aging-Related disease Risks. J Adv Res. doi:10.1016/j.jare.2026.05.022
2. Please explain the key elements of your study design and the use of the available datasets that make your study an original scientific contribution
Key Elements of Study Design<br>Population: The participants was derived from the 2017–2018 wave of the Chinese Longitudinal Healthy Longevity Survey (CLHLS). We restricted our analytical sample to community-dwelling older adults aged 65 years and less than 100 years who had complete data on MMSE scale and childhood hunger status.<br>Exposure: The primary exposure was self-reported childhood hunger, ascertained from the life-history section of the CLHLS questionnaire. Participants were asked whether they "often went to bed hungry as a child" (yes/no). In additional, arm length and knee height of elderly are common anthropometric measurements, which are also accurate representation of nutritional deprivation in childhood. Therefore, arm length or knee height is classified into

abnormal and normal group according to whether that is below 10th percentile stratified by sex based on previous study in sensitivity analysis.

Outcomes: The outcome of interest was cognitive impairment in late life. Cognitive function was assessed using the validated Chinese version of the Mini-Mental State Examination (MMSE), which was administered by trained interviewers in the 2017–2018 survey. Participants with MMSE scores below the education-adjusted cutoff (i.e., ≤17 for illiterate, ≤20 for primary school, and ≤24 for middle school and above) were defined as cases of cognitive impairment, following standard CLHLS practices. Moreover, participants with MMSE scores below 18 point were defined as case group in sensitivity analysis.

Covariates and Confounding Control: the following variables are regarded as candidate confounders: sex (male or female), race (Han or minority), the region of birth (eastern region, the central region, northeast region or western region), the residence of birth (urban or rural), the decade of birth (1910s-1920s, 1930s, 1940s or 1950s), main source of water drank in childhood (nature water or tap water), drink in childhood (yes or no), physical labor in childhood (yes or no), received schooling in childhood (yes or no), mother received schooling (yes or no), father received schooling (yes or no), father had stable job in participants' childhood (yes or no), lost mother in childhood (yes or no), and lost father in childhood (yes or no).

Data set: The CLHLS was a longitudinal healthy longevity survey among Chinese people aged 65 years and over. The CLHLS included seven subsequent waves of surveys (2000, 2002, 2005, 2008-2009, 2011-2012, 2014, 2017-2018) conducted in 22 provinces, municipalities and autonomous region, in addition to the first survey in 1998. The CLHLS datasets are publicly accessible at no cost through the official data repository after completing an online registration and application process. The data can be obtained from: https://opendata.pku.edu.cn/dataverse/CHADS. Among the15,874 participants in the cross-sectional survey data of the 2017–2018 wave released on the official CLHLS website, over 12,000 were interviewed during the 2017–2018 survey period, while the remaining participants were derived from previous waves.

3. Please list all publications that addressed similar research questions in the same dataset and indicate where you cited them in your paper

[1] Zhang Z, Gu D, Hayward MD. Childhood nutritional deprivation and cognitive impairment among older Chinese people. Soc Sci Med. 2010;71(5):941-949. doi:10.1016/j.socscimed.2010.05.013

[2] Dong H, Du C, Wu B, Wu Q. Multi-State Analysis of the Impact of Childhood Starvation on the Healthy Life Expectancy of the Elderly in China. Front Public Health. 2021;9:690645. doi:10.3389/fpubh.2021.690645

[3] Hu M, Shu X, Yu G, Wu X, Välimäki M, Feng H. A Risk Prediction Model Based on Machine Learning for Cognitive Impairment Among Chinese Community-Dwelling Elderly People With Normal Cognition: Development and Validation Study. J Med Internet Res. 2021;23(2):e20298. doi:10.2196/20298

[4] Ren L, Hu F, Walsh S, et al. Healthy lifestyle factors outweigh influence of APOE genetic risk on extending cognitively healthy life expectancy among Chinese older adults: evidence from a nationwide cohort study. Alzheimers Dement. 2025;21(4):e70090. doi:10.1002/alz.70090

[5] Wang X, Hu X, Chen F, et al. Association between childhood hunger and depression or anxiety in later life: Insights from two case-control studies. Psychiatry Res. 2025;348:116475. doi:10.1016/j.psychres.2025.116475

The first and second reference listed above was cited in the Methods section to describe the measurement of exposure, which included self-reported childhood hunger (i.e., often going to bed hungry during childhood), and the first reference also suggested the measurements of knee height and arm length, and the criteria for determining nutritional deprivation. In addition, this same reference was also cited in the Introduction, where we pointed out its limitations, which served as the rationale for conducting our present case-control study.

References 3–4 were used as the basis for dichotomizing the MMSE score at a cutoff of 18 to define the case and control groups, and they were accordingly cited in the Methods section under the description of outcome measurement.

Reference 5 is a concurrently conducted and already published study by our group on childhood hunger and late-life mental health; however, it was not cited in the present paper.

4. Please explain how you addressed multiple testing through an appropriately rigorous statistical threshold and indicate this in the methods section

Not applicable

5. Please declare to what extent have AI chatbots been used in developing your paper and to which parts of the paper did they contribute

We declare that no AI chatbots or large language models were used at any stage of developing this paper. They did not contribute to the study design, data analysis, interpretation, writing, or any other part of the manuscript. All work was performed solely by the authors.

Table S2. The names, labels and transformation of variables in the study

Names of variables	Labels	Source or definitions
**Childhood hunger**	1= yes, 0=no	Did you frequently go to bed hungry as a child?
**Cognitive impairment**	1= yes, 0=no	The mini-mental state examination scale (Chinese version) score was less than 18, 21 and 25 or not when the years of schooling was 0, 1-6 and more than 6, respectively.
**Age**	Current Age	Validated age at the time of survey
**Sex**	1=male, 2=female	Sex of participants
**Race**	1=Han, 2=Minority	Ethnic group (Han, Hui, Zhuang, Yao, Korea, Manchu, Mongolia, Other)
**The region of birth**	1=Eastern region, 2=The central region, 3=Northeast region, 4=Western region	Which province were you born in? Eastern region: Beijing, Tianjin, Hebei, Shandong, Jiangsu, Shanghai, Zhejiang, Fujian, Guangdong and Hainan, Hong Kong, Macao and Taiwan; The central region: Shanxi, Henan, Hubei, Anhui, Jiangxi and Hunan; Northeast region: Liaoning, Jilin, Heilongjiang and inner Mongolia; Western region: Shaanxi, Ningxia, Gansu, Chongqing, Sichuan, Qinghai, Guizhou, Yunnan, Guangxi
**The residence of birth**	1=urban, 2=rural	Was the place of birth an urban area or a rural area at time of birth?
**The decade of birth**	1=1910s-1920s, 2=1930s, 3=1940s, 4=1950s	Validated birth year: 1910-1959
**Main source of water drank in childhood**	1= nature water, 2=tap water	Main source of water drank during childhood. Nature water from a well, river or lake, spring, pond or pool; tap water
**Drink in childhood**	1= yes, 0=no	How old were you when you began to drink alcohol? If the age when participants began drinking was less than13 years, participants were considered drinking in childhood. If the age when participants began drinking was more than12 years, participants were not considered drinking in childhood.
**Physical labour in childhood**	1= yes, 0=no	How old were you when you began to do physical labor? If the age when participants began participate

		in physical labor was less than13 years, participants were considered participate in physical labor in childhood. If the age when participants began participate in physical labor was more than12 years, participants were not considered participate in physical labor in childhood.
**Received schooling in childhood**	1= yes, 0=no	How many years did you attend school?<br>If participants' years of schooling were equal to 0, so that participants did not receive schooling in childhood. If participants' years of schooling were equal or more than 1, so that participants received schooling in childhood.
**Mother received schooling**	1= yes, 0=no	How many years did your mother attend school?<br>If years of schooling of participants' mother were equal to 0, so that they did not receive schooling. If years of schooling of participants' mother were equal or more than 1, so that they received schooling.
**Father received schooling**	1= yes, 0=no	How many years of schooling did your father receive?<br>If years of schooling of participants' father were equal to 0, so that they did not receive schooling. If years of schooling of participants' father were equal or more than 1, so that they received schooling.
**Father had stable job in participants' childhood**	1= yes, 0=no	What was your father's occupation when you are a child?<br>If the occupation was professional and technical personnel, governmental, institutional or managerial personnel, commercial, service or industrial worker, military personnel and others, so that participants' father had stable job, otherwise participants' father had not stable job.
**Lost mother in childhood**	1= yes, 0=no	If your mother is dead, how old were you when she died?<br>If the participants' age at mother's death were less than 13, so that they lost mother in childhood. If the participants' age at mother's death were more than 12, so that they did not lost mother in childhood.
**Lost father in**	1= yes, 0=no	If your father is dead, how old were you when

**childhood**		he died?<br>If the participants' age at father's death were less than 13, so that they lost father in childhood. If the participants' age at father's death were more than 12, so that they did not lost father in childhood.
**The Mini-Mental State Examination**		
Orientation		
What time of day is it right now<br>What is the animal year of this year<br>What is the date of the mid-autumn festival<br>What is the season right now<br>What is the name of this county or district	0=wrong, 1=correct, 8=unable to answer	If the answer is "wrong" or "unable to answer", the score is 0; if the answer is "correct", the score is 1
Name the things that can be eaten	Score 1 for 1 correct answer, score 7 for 7 or more answers	
Recall		
Name the "table" correctly<br>Name the "apple" correctly<br>Name the "clothes" correctly	0=wrong, 1=correct, 8=unable to answer	If the answer is "wrong" or "unable to answer", the score is 0; if the answer is "correct", the score is 1
Attention and calculation		
20–3 =?<br>20–3-3 =?<br>20–3-3-3 =?<br>20–3–3-3-3-3 =?<br>20–3–3-3-3-3-3 =?<br>Copy the figure on the card	0=wrong, 1=correct, 8=unable to answer	If the answer is "wrong" or "unable to answer", the score is 0; if the answer is "correct", the score is 1
Delayed recall		
Repeat "table, apple, clothes" as remembered in the "responsiveness section"	0=wrong, 1=correct, 8=unable to answer	If the answer is "wrong" or "unable to answer", the score is 0; if the answer is "correct", the score is 1

Language		
Name the object the investigator is pointing to as "pen"	0=wrong, 1=correct, 8=unable to answer	If the answer is "wrong" or "unable to answer", the score is 0; if the answer is "correct", the score is 1
Name the "watch" to which the investigator is referring		
Repeat the assigned sentence from the investigator		
Ask the respondent to hold the paper in their right hand		
Ask the respondent to fold the paper in half		
Ask the respondent to place the paper on the floor		

Table S3. The description of variables with missing value

Variables	Frequency (*n*)	Proportion (%)
**Got adequate medical treatment in childhood**	7591	81.65
**Smoke in childhood**	6349	68.29
**Exercise in childhood**	5343	57.47
**Physical labour in childhood**	2535	27.27
**Lost father in childhood**	1915	20.60
**Lost mother in childhood**	1905	20.49
**Mother received schooling**	1156	12.43
**Father received schooling**	522	5.61
**Main source of water drank in childhood**	390	3.98
**The region of birth**	335	3.60
**Father had stable job in participants' childhood**	262	2.82
**Drink in childhood**	197	2.12
**Race**	195	2.10
**The residence of birth**	192	2.07
**Received schooling in childhood**	170	1.83

Table S4. Detailed information about hierarchical regression analyses

Models	Variables	β	SE	OR (95% CI)	*P*-value
**Model 1**	Childhood hunger				
	No	ref			
	Yes	0.364	0.0749	1.440 (1.243-1.667)	<0.001
**Model 2**	Childhood hunger				
	No	ref			
	Yes	0.211	0.081	1.235 (1.053-1.449)	0.009
	The decade of birth				
	1910s-1920s	ref			
	1930s	-1.461	0.081	0.232 (0.198-0.272)	<0.001
	1940s	-2.474	0.122	0.084 (0.066-0.107)	<0.001
	1950s	-2.966	0.244	0.052 (0.032-0.083)	<0.001
	Sex				
	Male	ref			
	Female	0.186	0.075	1.204 (1.039-1.395)	<0.001
	Received schooling in childhood				
	No	ref			
	Yes	-0.273	0.079	0.761 (0.652-0.889)	<0.001
**Model 3**	Childhood hunger				
	No	ref			
	Yes	0.211	0.083	1.234 (1.049-1.453)	0.011
	The decade of birth				
	1910s-1920s	ref			
	1930s	-1.459	0.0811	0.233 (0.198-0.273)	<0.001
	1940s	-2.474	0.122	0.084 (0.066-0.107)	<0.001
	1950s	-2.967	0.2439	0.052 (0.032-0.083)	<0.001
	Sex				
	Male	ref			
	Female	0.1908	0.0756	1.210 (1.044-1.403)	0.012
	Received schooling in childhood				
	No	ref			
	Yes	-0.238	0.0824	0.788 (0.671-0.927)	0.004
	Mother received schooling				
	No	ref			
	Yes	0.305	0.189	1.356 (0.936-1.966)	0.107
	Father received schooling				
	No	ref			
	Yes	-0.403	0.1126	0.668 (0.536-0.833)	<0.001
	Father had a stable job in participants' childhood				
	No	ref			
	Yes	-0.157	0.0999	0.855(0.702-1.040)	0.012

**Model 4**	Childhood hunger				
	No	ref			
	Yes	0.206	0.083	1.229 (1.044-1.447)	0.013
	The decade of birth				
	1910s-1920s	ref			
	1930s	-1.461	0.081	0.232 (0.198-0.272)	<0.001
	1940s	-2.473	0.122	0.084 (0.066-0.107)	<0.001
	1950s	-2.965	0.244	0.052 (0.032-0.083)	<0.001
	Sex				
	Male	ref			
	Female	0.191	0.076	1.210 (1.043-1.403)	0.012
	Received schooling in childhood				
	No	ref			
	Yes	-0.236	0.082	0.790 (0.672-0.928)	0.004
	Mother received schooling				
	No	ref			
	Yes	0.307	0.189	1.359 (0.938-1.970)	0.105
	Father received schooling				
	No	ref			
	Yes	-0.4032	0.113	0.668 (0.536-0.833)	<0.001
	Father had a stable job in participants' childhood				
	Yes	ref			
	No	-0.154	0.100	0.858 (0.705-1.043)	0.124
	Lost mother in childhood				
	No	ref			
	Yes	0.145	0.109	1.156 (0.933-1.432)	0.185

SE–standard error, OR – odds ratio, CI – confidence interval, ref – reference

Table S5. The comparison of characteristics between case group and control group after 1:1 propensity score matching*

Variables	Total (*n*=2,216)	Cognitive impairment		χ^2/*t*/*Z*	*P*-value
		No (n=1,108)	Yes (n=1,108)		
**Childhood hunger**				5.033	0.025
Yes	1652 (74.55)	803 (72.47)	849 (76.62)		
No	564 (25.45)	305 (27.53)	259 (23.38)		
**Matching Variables**					
**The decade of birth**				0.000	1.000
1910s-1920s	1546 (69.77)	773 (69.77)	773 (69.77)		
1930s	468 (21.12)	234 (21.12)	234 (21.12)		
1940s	166 (7.49)	83 (7.49)	83 (7.49)		
1950s	36 (1.62)	18 (1.62)	18 (1.62)		
**Sex**				0.000	1.000
Male	902 (40.70)	451 (40.70)	451 (40.70)		
Female	1314 (59.30)	657 (59.30)	657 (59.30)		
**Received schooling in childhood**				0.008	0.930
No	1374 (62.00)	686 (61.91)	688 (62.09)		
Yes	842 (38.00)	422 (38.09)	420 (37.91)		
**Mother received schooling**				0.013	0.909
No	2137 (96.44)	1069 (96.48)	1068 (96.39)		
Yes	79 (3.56)	39 (3.52)	40 (3.61)		
**Father received schooling**				0.065	0.798
No	1936 (87.36)	970 (87.55)	966 (87.18)		
Yes	280 (12.64)	138 (12.45)	142 (12.82)		
**Father had stable job in participants' childhood**				0.000	1.000
Yes	394 (17.78)	197 (17.78)	197 (17.78)		
No	1822 (82.22)	911 (82.22)	911 (82.22)		
**Lost mother in childhood**				0.165	0.685
No	1970 (88.90)	988 (89.17)	982 (88.63)		
Yes	246 (11.10)	120 (10.83)	126 (11.37)		
**Non-matching Variables**					
**Age, x̄ (SD)**	89.33±7.31	88.77±7.24	89.90±7.35	-3.629	<0.001
**Race**				0.473	0.492
Han	2107 (95.08)	1050 (94.77)	1057 (95.40)		
Minority	109 (4.92)	58 (5.23)	51 (4.60)		
**The region of birth**				0.324	0.955
Eastern region	1034 (46.66)	514 (46.39)	520 (46.93)		
The central region	524 (23.65)	259 (23.38)	265 (23.92)		
Northeast region	90 (4.06)	46 (4.15)	44 (3.97)		
Western region	568 (25.63)	289 (26.08)	279 (25.18)		
**The residence of birth**				0.013	0.910

Variables	Total (*n*=2,216)	Cognitive impairment		$\chi^2/t/Z$	*P*-value
		No (n=1,108)	Yes (n=1,108)		
Urban	376 (16.97)	187 (16.88)	189 (17.06)		
Rural	1840 (83.03)	921 (83.12)	919 (82.94)		
**Main source of water drank in childhood**				0.576	0.448
Nature water	2026 (91.43)	1018 (91.88)	1008 (90.97)		
Tap water	190 (8.57)	90 (8.12)	100 (9.03)		
**Drink in childhood**				0.067	0.796
No	2201 (99.32)	1101 (99.37)	1100 (99.28)		
Yes	15 (0.68)	7 (0.63)	8 (0.72)		
**Physical labour in childhood**				2.867	0.090
No	1923 (86.78)	948 (85.56)	975 (88.00)		
Yes	293 (13.22)	160 (14.44)	133 (12.00)		
**Lost father in childhood**				1.412	0.235
No	1921 (86.69)	951 (85.83)	970 (87.55)		
Yes	295 (13.31)	157 (14.17)	138 (12.45)		
**MMSE, Mdn (Q_1, Q_3)**	20.00 (13.00, 27.00)	27.00 (23.00,29.00)	13.00 (7.00, 17.00)	38.997	<0.001

Mdn – median, MMSE – Mini-Mental State Examination, SD – standard deviation, $\bar{x}$ – mean

*Presented as n (%) unless specified otherwise.

Table S6. The association between arm length or knee height and cognitive impairment based on multivariable unconditional logistic regressions

Variables	β	SE	OR (95% CI)	*P*-value
**Arm length or knee height**				
Normal	ref			
Abnormal	0.301	0.090	1.351 (1.132-1.612)	0.001
**The decade of birth**				
1910s-1920s	ref			
1930s	-1.446	0.082	0.236 (0.201-0.277)	<0.001
1940s	-2.460	0.124	0.085 (0.067-0.109)	<0.001
1950s	-2.916	0.244	0.054 (0.034-0.087)	<0.001
**Sex**				
Male	ref			
Female	0.183	0.076	1.200 (1.034-1.394)	0.017
**Received schooling in childhood**				
No	ref			
Yes	-0.273	0.082	0.761 (0.648-0.894)	0.001
**Mother received schooling**				
No	ref			
Yes	0.295	0.189	1.343 (0.927-1.946)	0.119
**Father received schooling**				
No	ref			
Yes	-0.392	0.113	0.676 (0.541-0.843)	0.001
**Father had stable job in participants' childhood**				
Yes	ref			
No	-0.096	0.100	0.908 (0.747-1.105)	0.336
**Lost mother in childhood**				
No	ref			
Yes	0.164	0.110	1.178 (0.95-1.461)	0.136

SE–standard error, OR – odds ratio, CI – confidence interval, ref – reference

Table S7. The association between arm length or knee height and cognitive impairment based on CCW-TMLE*

Causal effect measures	Estimate	95% CI	*P*-value
Risk ratio	1.250	1.096-1.426	<0.001
Odds ratio	1.292	1.109-1.504	0.001
Risk difference (‰)	28.37	10.40-46.34	0.002

CCW-TMLE – case-control weighted target maximum likelihood estimation, CI – confidence interval

*Confounders: the decade of birth, sex, received schooling in childhood, mother received schooling, father received schooling, father had a stable job in participants' childhood and lost mother in childhood.

Table S8. The multivariable unconditional logistic regression based on dataset imputed by random forest algorithm

Variables	β	SE	OR (95% CI)	*P*-value
**Childhood hunger**				
No	ref			
Yes	0.212	0.083	1.236 (1.051-1.455)	0.011
**The decade of birth**				
1910s-1920s	ref			
1930s	-1.434	0.081	0.238 (0.203-0.279)	<0.001
1940s	-2.475	0.123	0.084 (0.066-0.107)	<0.001
1950s	-2.909	0.238	0.055 (0.034-0.087)	<0.001
**Sex**				
Male	ref			
Female	0.171	0.076	1.187 (1.023-1.376)	0.024
**Received schooling in childhood**				
No	ref			
Yes	-0.213	0.083	0.809 (0.688-0.951)	0.010
**Mother received schooling**				
No				
Yes	0.396	0.100	1.485 (1.222-1.806)	<0.001
**Father received schooling**				
No				
Yes	-0.282	0.101	0.754 (0.619-0.920)	0.005
**Father had stable job in participants' childhood**				
Yes	ref			
No	-0.104	0.1015	0.901 (0.738-1.099)	0.305
**Lost mother in childhood**				
No	ref			
Yes	0.477	0.080	1.611 (1.376-1.885)	<0.001

SE–standard error, OR – odds ratio, CI – confidence interval, ref – reference

Table S9. The CCW-TMLE method based on dataset imputed by random forest algorithm*

Causal effect measures	Estimate	95% CI	*P*-value
Risk ratio	1.182	1.060-1.317	0.003
Odds ratio	1.244	1.081-1.432	0.003
Risk difference (‰)	39.44	14.93-63.94	0.002

CCW-TMLE – case-control weighted target maximum likelihood estimation, CI – confidence interval

*Confounders: the decade of birth, sex, received schooling in childhood, mother received schooling, father received schooling, father had a stable job in participants' childhood and lost mother in childhood.

Table S10. The multivariable unconditional logistic regression adjusting furtherly an additional confounder

Variables	β	SE	OR (95% CI)	*P*-value
**Childhood hunger**				
No	ref			
Yes	0.212	0.083	1.236 (1.049-1.455)	0.011
**The decade of birth**				
1910s-1920s	ref			
1930s	-1.458	0.081	0.233 (0.198-0.273)	<0.001
1940s	-2.472	0.122	0.084 (0.066-0.107)	<0.001
1950s	-2.968	0.244	0.051 (0.032-0.083)	<0.001
**Sex**				
Male	ref			
Female	0.186	0.076	1.204 (1.038-1.397)	0.014
**Received schooling in childhood**				
No	ref			
Yes	-0.241	0.083	0.786 (0.669-0.924)	0.004
**Mother received schooling**				
No	ref			
Yes	0.307	0.189	1.359 (0.937-1.969)	0.106
**Father received schooling**				
No	ref			
Yes	-0.410	0.113	0.664 (0.532-0.828)	<0.001
**Father had stable job in participants' childhood**				
Yes	ref			
No	-0.161	0.100	0.851 (0.700-1.036)	0.107
**Lost mother in childhood**				
No	ref			
Yes	0.175	0.110	1.192 (0.960-1.479)	0.111
**Lost father in childhood**				
No	ref			
Yes	-0.235	0.103	0.791 (0.647-0.968)	0.023

SE–standard error, OR – odds ratio, CI – confidence interval, ref – reference

Table S11. The CCW-TMLE method adjusting furtherly an additional confounder*

Causal effect measure	Estimate	95% CI	*P*-value
Risk ratio	1.198	1.069-1.342	0.002
Odds ratio	1.266	1.094-1.465	0.002
Risk difference (‰)	42.42	17.08-67.76	0.001

CCW-TMLE – case-control weighted target maximum likelihood estimation, CI – confidence interval

*Confounders: the decade of birth, sex, received schooling in childhood, mother received schooling, father received schooling, father had a stable job in participants' childhood, lost mother in childhood and lost father in childhood.

Table S12. The CCW-TMLE method based on a target population prevalence of cognitive impairment set to 22.41%*

Causal effect measures	Estimate	95% CI	*P*-value
Risk ratio	1.179	1.052-1.322	0.005
Odds ratio	1.232	1.068-1.422	0.004
Risk difference (‰)	34.73	11.74-57.73	0.003

CCW-TMLE – case-control weighted target maximum likelihood estimation, CI – confidence interval

*Confounders: the decade of birth, sex, received schooling in childhood, mother received schooling, father received schooling, father had a stable job in participants' childhood and lost mother in childhood.

Table S13. The multivariable unconditional logistic regression using an MMSE cutoff of 18 to redefine cognitive impairment

Variables	β	SE	OR (95% CI)	*P*-value
**Childhood hunger**				
No	ref			
Yes	0.269	0.095	1.309 (1.086-1.579)	0.005
**The decade of birth**				
1910s-1920s	ref			
1930s	-1.580	0.090	0.206 (0.172-0.246)	< 0.001
1940s	-2.924	0.170	0.054 (0.039-0.075)	< 0.001
1950s	-3.557	0.384	0.029 (0.013-0.061)	< 0.001
**Sex**				
Male	ref			
Female	0.255	0.084	1.29 (1.093-1.522)	0.003
**Received schooling in childhood**				
No	ref			
Yes	-0.786	0.095	0.455 (0.378-0.549)	< 0.001
**Mother received schooling**				
No	ref			
Yes	0.321	0.236	1.378 (0.867-2.191)	0.175
**Father received schooling**				
No	ref			
Yes	-0.510	0.138	0.601(0.458-0.787)	< 0.001
**Father had stable job in participants' childhood**				
Yes	ref			
No	0.015	0.119	1.015 (0.804-1.281)	0.902
**Lost mother in childhood**				
No	ref			
Yes	0.264	0.117	1.302 (1.036-1.638)	0.024

SE–standard error, OR – odds ratio, CI – confidence interval, ref – reference

Table S14. The CCW-TMLE method using an MMSE cutoff of 18 to redefine cognitive impairment*

**Causal effect measures**	**Estimate**	**95% CI**	***P*-value**
Risk ratio	1.208	1.066-1.370	0.003
Odds ratio	1.264	1.084-1.474	0.003
Risk difference (‰)	36.56	13.65-59.48	0.002

CCW-TMLE – case-control weighted target maximum likelihood estimation, CI – confidence interval

*Confounders: the decade of birth, sex, received schooling in childhood, mother received schooling, father received schooling, father had a stable job in participants' childhood and lost mother in childhood.

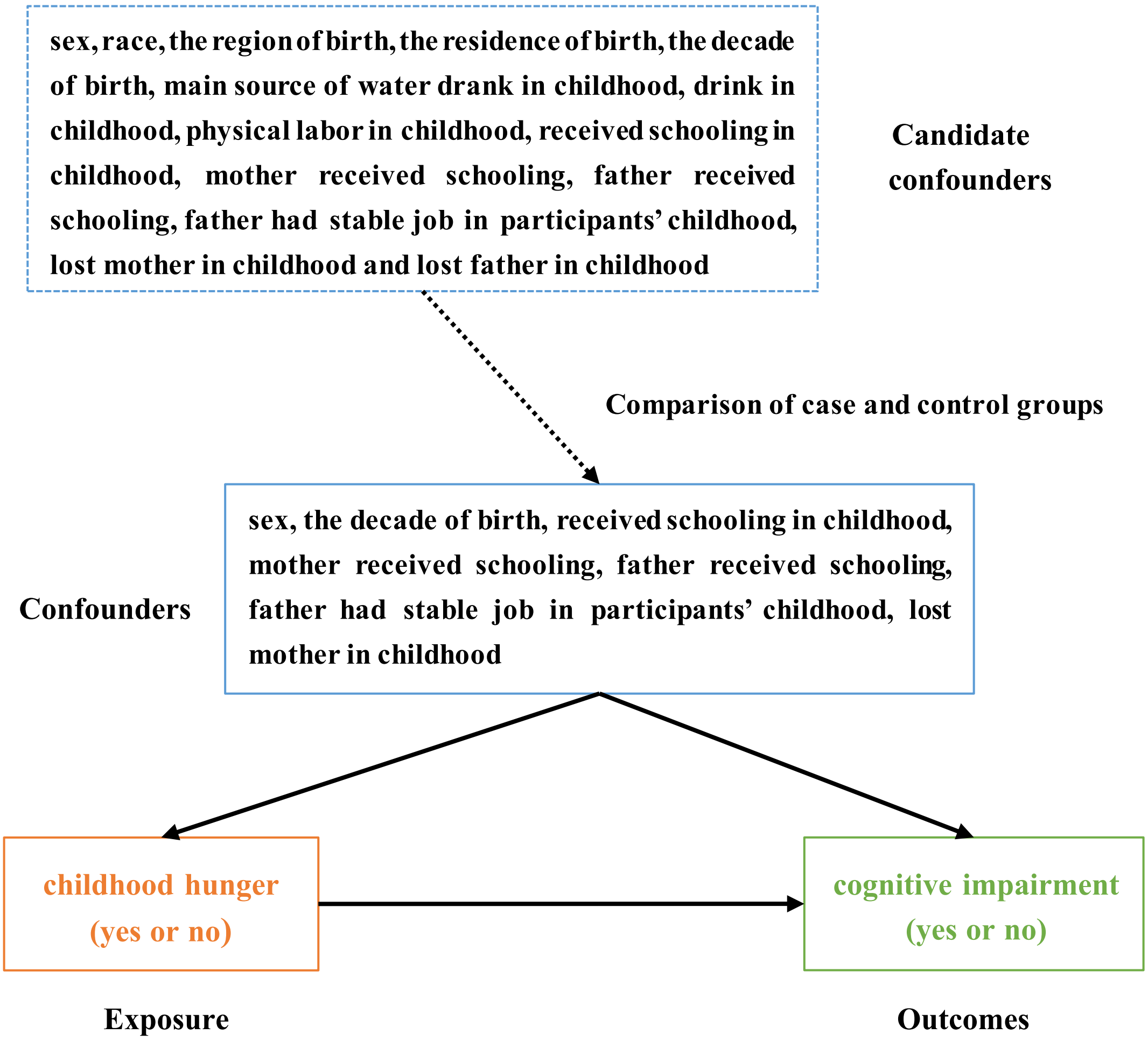

Figure S1. The simplified causal directed acyclic graphs showed the relationship and directions of path among exposure, confounders and outcome
